# Dietary exposure to creatine-precursor amino acids in the general population

**DOI:** 10.1007/s00726-025-03460-7

**Published:** 2025-05-24

**Authors:** David Nedeljkovic, Sergej M. Ostojic

**Affiliations:** 1 Applied Bioenergetics Lab, Faculty of Sport and Physical Education, University of Novi Sad, Novi Sad, Serbia; 2 Department of Nutrition and Public Health, University of Agder, Kristiansand, Norway; 3 Faculty of Health Sciences, University of Pécs, Pécs, Hungary

**Keywords:** Creatine, Metabolism, NHANES, Amino acids, Nutrition, Precursor

## Abstract

**Background:**

Creatine is a semi-essential nutrient that plays a critical role in energy metabolism, with dietary intake and endogenous synthesis contributing to overall creatine availability. While dietary creatine intake has been studied extensively, limited data exist on the dietary exposure to its precursor amino acids—glycine, arginine, and methionine—and their contribution to endogenous creatine synthesis. This study aimed to assess the dietary intake of these precursors in U.S. children and adults using data from the Third National Health and Nutrition Examination Survey (NHANES III) and to compare endogenous creatine synthesis with direct dietary creatine intake.

**Methods:**

We analyzed NHANES III dietary recall data from 29,945 individuals aged 2 years and older. Intakes of glycine, arginine, methionine, and creatine were calculated per kilogram of body weight. The contribution of precursor amino acids to endogenous creatine synthesis was estimated using established metabolic conversion factors.

**Results:**

The mean daily intakes of glycine, arginine, methionine, and creatine were 59.6 ± 0.4 mg/kg, 77.2 ± 0.5 mg/kg, 31.9 ± 0.2 mg/kg, and 15.5 ± 0.1 mg/kg, respectively. Estimated endogenous creatine synthesis from precursor amino acids was significantly greater than dietary creatine intake across all age groups (*P* < 0.01), with precursor-derived creatine production averaging 41.9 ± 0.3 mg/kg body weight per day, approximately 2.7 times higher than dietary creatine intake. Creatine precursor availability declined with age, with the lowest values observed in individuals aged ≥ 65 years.

**Conclusion:**

This study provides the first comprehensive evaluation of total creatine availability in a representative U.S. population, highlighting the predominance of endogenous synthesis over direct dietary intake. These findings suggest that creatine metabolism is largely dependent on precursor amino acid intake and that certain populations, particularly older adults, may be at higher risk for reduced creatine availability. Future research should explore the physiological implications of these findings and potential dietary interventions to optimize creatine status across the lifespan.

## Introduction

Creatine (methylguanidoacetic acid) is a non-proteinogenic amino acid derivative and is considered a semi-essential nutrient in human nutrition (Ostojic and Forbes 2022). It functions as a rapid energy buffer, primarily in tissues with high energy demands, such as skeletal muscle, the brain, and the heart (Wyss and Kaddurah-Daouk 2000). Maintaining adequate creatine levels is essential for optimal physical performance, metabolic health, and disease prevention (Wu 2020; Ostojic 2022). Although the human body can endogenously synthesize creatine, dietary intake may be necessary for individuals with higher creatine demands or insufficient endogenous production. A classical dogma of creatine metabolism is that approximately half of the daily creatine requirement is obtained from animal-based dietary sources, while the remaining half is synthesized endogenously from three amino acids: glycine and arginine (semi-essential amino acids) and methionine (an essential amino acid) (Brosnan and Brosnan 2016). While several studies have quantified dietary creatine intake from food sources (Bakian et al. 2020; Korovljev et al. 2021), limited data are available on dietary exposure to creatine-precursor amino acids in the general population (Institute of Medicine 2005), particularly regarding the proportion of these precursors utilized for endogenous creatine synthesis. Understanding the intake of these precursors is crucial for assessing the relative contribution of endogenous synthesis versus direct dietary creatine intake. Therefore, the primary objective of this study was to assess the dietary intake of glycine, arginine, and methionine among U.S. children and adults using data from the Third National Health and Nutrition Examination Survey (NHANES III). Additionally, our study aimed to compare endogenous creatine synthesis from creatine-precursor amino acids with dietary creatine intake within the same population. This comparison may offer valuable insights into the relative contributions of these sources, thereby improving our understanding of creatine metabolism and its potential dietary determinants.

## Methods

The NHANES is a large-scale, nationally representative health survey conducted in the United States since the early 1960s. Its primary objective is to assess the health and nutritional status of the non-institutionalized U.S. civilian population through a combination of interviews, physical examinations, and laboratory tests. NHANES III, conducted between 1988 and 1994, was a nationwide probability sample of 39,695 individuals aged 2 months and older. This survey provided comprehensive data on dietary intake, biochemical markers, chronic diseases, and lifestyle factors across diverse demographic groups. Detailed methodology and survey design information for NHANES III can be found elsewhere (National Center for Health Statistics, 2025). For this analysis, we extracted data from NHANES III for all participants aged 2 years and older who provided dietary intake information via a 24-hour food recall, as recorded in the Individual Food File database. The intakes of glycine, arginine, and methionine were directly obtained from individual food records, while creatine intake in individual foods was calculated based on previously established methods (Todorovic et al. 2022; Ostojic 2025). Nutrient intakes were aggregated across all reported foods for each participant, with the intake of each nutrient was quantified in relative amount (mg per kg body weight). In addition, adjusted estimates were calculated to determine the presumed amount of endogenous creatine synthesis from dietary glycine, arginine, and methionine (Brosnan et al. 2011). Specifically, the entire glycine molecule is incorporated into the creatine structure (100%), while 25% of the amidino groups from arginine and 40% of the methyl groups from *S*-adenosylmethionine, a direct metabolic derivative of methionine, also contribute to the synthesis process (see Table 1 for correction factors).

**Table 1:** Contribution of precursors to endogenous creatine synthesis per dietary exposure (DE)

Nutrient	Portion used for creatine synthesis	Adjusted contribution	Fraction
Glycine	100% (entire molecule)	DE × 1.00 × (75.1 / 131.1)	57.2%
Arginine	25% of amidino groups	DE × 0.25 × (43.1 / 131.1)	8.2%
Methionine	40% of methyl groups	DE × 0.40 × (15.0 / 131.1)	4.6%

*Note*: Molecular weights: glycine 75.1 g/mol, creatine 131.1 g/mol, amidino group 43.1 g/mol, methyl group 15.0 g/mol

All analyses were conducted for the total sample as well as subsamples stratified by gender and age categories. Approval to conduct NHANES III was granted by the National Center for Health Statistics (NCHS) Research Ethics Review Board. To compare dietary intakes across demographic strata, we employed one-way ANOVA for multiple group comparisons. A Tukey post-hoc test was then used to identify significant differences between individual sample pairs. All values are presented as mean ± SE. Data were analyzed using SPSS Version 24 (IBM, Armonk, NY, USA), with statistical significance set at *P* ≤ 0.05.

## Results

After excluding duplicate entries, a total of 29,945 respondents from the NHANES III cohort (mean age 36.0 ± 25.6 years, with 51.2% female) provided valid data on dietary intake for all four nutrients. The mean daily dietary intakes per kg body weight for glycine, arginine, methionine, and creatine across the entire sample were as follows: 59.6 ± 0.4 mg (95% confidence interval [CI], 58.7 to 60.4), 77.2 ± 0.5 mg (95% CI, 76.2–78.3), 31.9 ± 0.2 mg (95% CI, 31.5–32.4), and 15.5 ± 0.1 mg (95% CI, 15.3–15.9), respectively. The dietary intakes of these nutrients across age and gender categories are presented in Table 2. One-way ANOVA indicated a significant difference in dietary glycine, arginine, and methionine intake across age subgroups (*P* < 0.001). Post hoc analyses revealed significant differences between all individual sample pairs (*P* < 0.05), except for the comparisons between the 13–17 years and 18–64 years age groups in the total sample and gender-specific subsamples. Similar results were observed for dietary creatine intake across age subgroups (*P* < 0.001), with post hoc analyses revealing significant differences between most individual sample pairs (*P* < 0.05). Exceptions included the comparisons between the 13–17 years and 18–64 years age groups, as well as between the 13–17 years and ≥ 65 years age groups in the total sample. In the male subsample, no significant differences were found between the 5–12 years and 18–64 years age groups, the 13–17 years and 18–64 years age groups, and the 13–17 years and ≥ 65 years age groups. Similarly, in the female subsample, no significant differences were observed between the 13–17 years and 18–64 years age groups or between the 13–17 years and ≥ 65 years age groups.

**Table 2:** Daily intakes (mg/kg body weight) of creatine-precursor amino acids and creatine across age subgroups in NHANES III (*n* = 29,945). Values are mean ± se

	2–4 years	5–12 years	13–17 years	18–64 years	≥ 65 years	*P* *
*Glycine (mg/kg body weigt/day)*						
Total	107.9 ± 4.1	78.9 ± 1.5	58.1 ± 1.6 ^1^	60.8 ± 0.6 ^1^	48.1 ± 0.7	< 0.001
Male	109.8 ± 6.1	78.8 ± 2.2	63.1 ± 2.6 ^1^	68.1 ± 1.1 ^1^	49.3 ± 0.9	< 0.001
Female	105.7 ± 5.5	79.0 ± 2.0	53.0 ± 1.7 ^1^	54.2 ± 0.6 ^1^	46.9 ± 1.2	< 0.001
*Arginine (mg/kg body weigt/day)*						
Total	150.7 ± 5.3	105.1 ± 1.9	76.4 ± 2.0 ^1^	78.2 ± 0.8 ^1^	62.2 ± 0.7	< 0.001
Male	156.0 ± 7.8	105.1 ± 2.8	82.8 ± 3.4 ^1^	86.9 ± 1.3 ^1^	64.3 ± 1.1	< 0.001
Female	145.1 ± 7.0	105.0 ± 2.6	69.8 ± 2.1 ^1^	70.4 ± 0.8 ^1^	60.3 ± 1.0	< 0.001
*Methionine (mg/kg body weigt/day)*						
Total	59.8 ± 2.3	44.6 ± 0.8	32.4 ± 0.9 ^1^	32.1 ± 0.3 ^1^	25.4 ± 0.3	< 0.001
Male	60.5 ± 3.4	45.1 ± 1.2	35.3 ± 1.5 ^1^	35.6 ± 0.5 ^1^	26.2 ± 0.4	< 0.001
Female	58.9 ± 3.1	44.2 ± 1.1	29.3 ± 0.9 ^1^	29.1 ± 0.3 ^1^	24.6 ± 0.4	< 0.001
*Creatine (mg/kg body weigt/day)*						
Total	27.9 ± 1.5	19.8 ± 0.5	14.4 ± 0.5 ^1 2^	15.9 ± 0.2 ^1^	13.0 ± 0.2 ^2^	< 0.001
Male	28.5 ± 2.2	19.4 ± 0.8 ^1^	15.6 ± 0.8 ^2 3^	17.7 ± 0.3 ^1 2^	13.5 ± 0.2 ^3^	< 0.001
Female	27.2 ± 2.1	20.2 ± 0.7	13.2 ± 0.5 ^2 3^	14.3 ± 0.2 ^2^	12.4 ± 0.2 ^3^	< 0.001

*Note: *An asterisk (*) denotes statistical significance across age groups as assessed by one-way ANOVA. Superscript numbers indicate no significant difference (P > 0.05) between individual sample pairs sharing the same number

**Fig. 1:**
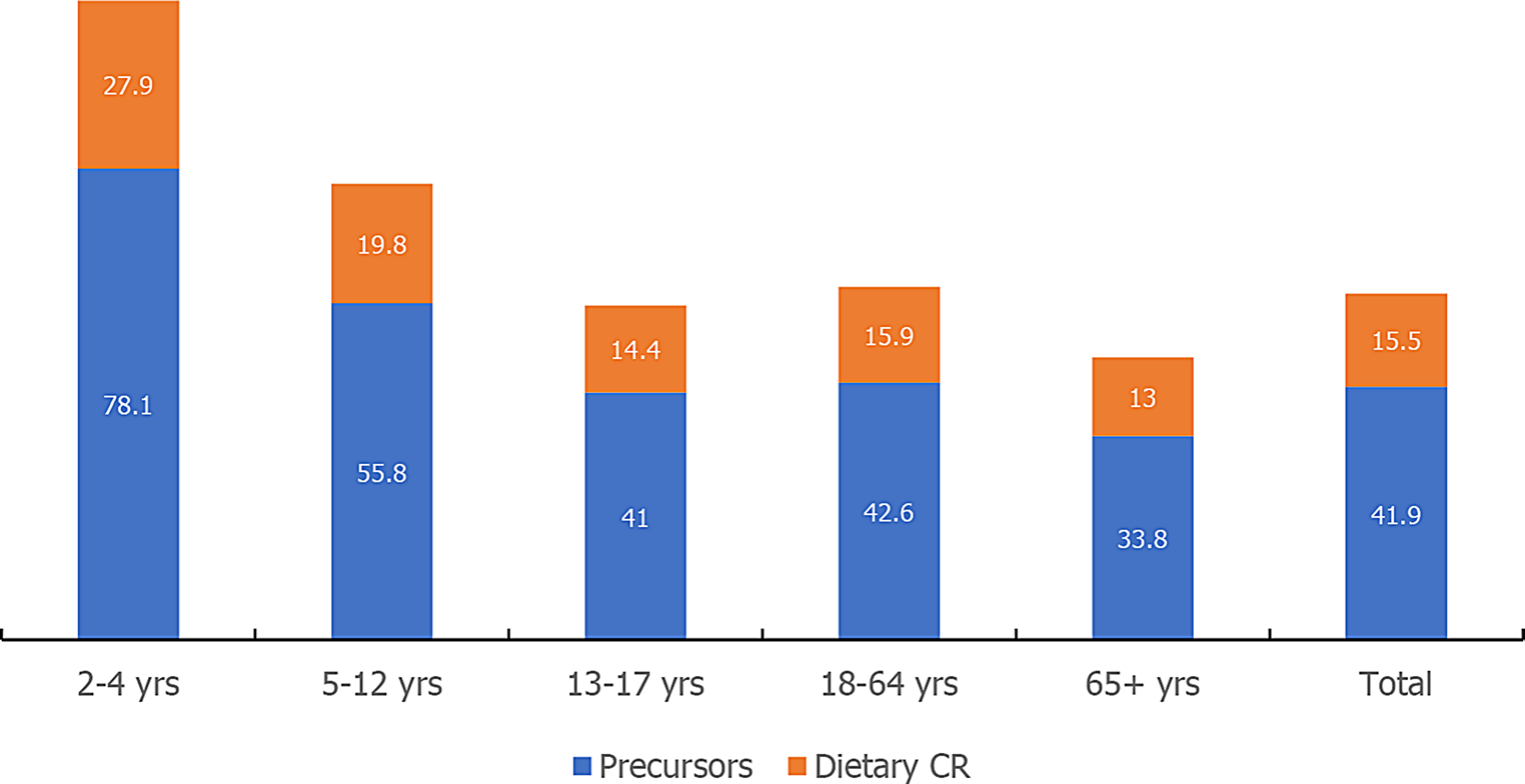
The presumed total creatine (CR) availability from food sources in mg per kg body weight per day across age groups

The estimated amount of creatine synthesized endogenously from glycine, arginine, and methionine in the total sample was 41.9 ± 0.3 mg/kg body weight per day (95% CI: 41.3–41.9; median: 27.7). When combined with dietary creatine intake, total creatine availability increased to 57.4 ± 0.4 mg/kg body weight per day (95% CI: 56.6–58.3; median: 37.2). The distribution of mean values for creatine availability across age groups is presented in Fig. 1. Notably, the estimated endogenous creatine synthesis was significantly higher than the creatine obtained from dietary sources across all age groups (*P* < 0.01).

## Discussion

To the best of our knowledge, this study is the first to comprehensively assess total creatine availability from dietary sources, considering both preformed creatine and its precursor amino acids—glycine, arginine, and methionine. Our findings indicate that the theoretical contribution of dietary precursors to endogenous creatine synthesis is, on average, approximately 2.7 times greater than the amount of creatine directly obtained from food. Moreover, this contribution declines with age, with the lowest values observed in individuals aged 65 years and older. While multiple factors influence endogenous creatine synthesis, our results suggest that certain populations may be more susceptible to reduced creatine availability.

Creatine is synthesized endogenously through a two-step enzymatic process primarily occurring in the kidneys, pancreas, and liver (Wyss and Kaddurah-Daouk 2000). In the first step, L-arginine: glycine amidinotransferase (AGAT) catalyzes the conversion of arginine and glycine into guanidinoacetate in the kidneys. This intermediate is then methylated in the liver by *S*-adenosyl-L-methionine: N-guanidinoacetate methyltransferase (GAMT), using *S*-adenosylmethionine as a methyl donor, to produce creatine. The principal regulatory enzyme in creatine biosynthesis is AGAT, which catalyzes the rate-limiting initial step of the pathway. Creatine exerts negative feedback on AGAT, suppressing its activity when intracellular creatine concentrations are adequate. In addition to this feedback mechanism, AGAT activity is modulated by various hormonal and nutritional factors, underscoring its central role in the regulation of creatine synthesis (Wyss and Kaddurah-Daouk 2000). Once synthesized, creatine is transported to target tissues, where it is phosphorylated into phosphocreatine, serving as a readily available energy reservoir for ATP resynthesis. Excess creatine undergoes degradation into creatinine, which is excreted in the urine to maintain homeostasis. Endogenous creatine synthesis is influenced by dietary intake of precursor amino acids, imposing a metabolic burden on their availability. Methionine and arginine may be particularly affected compared to glycine (Brosnan et al. 2011). However, the precise magnitude of endogenous creatine synthesis remains poorly characterized due to limited and largely indirect data. Evidence suggests that endogenous synthesis is upregulated in populations with minimal or no dietary creatine intake. Despite this compensatory response, it appears insufficient to fully offset the absence of dietary creatine, as overall creatine levels in these individuals remain lower than in omnivores (Delanghe et al. 1989). For example, skeletal muscle creatine concentrations in vegetarians are reported to be approximately 10% lower than in omnivores (Lukaszuk et al. 2002), suggesting that endogenous synthesis alone may not fully meet physiological demands. These findings highlight the significant contribution of endogenous synthesis relative to dietary intake, though the precise ratio between these sources remains uncertain.

Our study demonstrates that creatine precursors provide a substantial supply of the necessary building blocks for endogenous creatine production, surpassing direct dietary creatine intake in all age groups. The highest average ratio (2.8:1) was observed in non-adult populations, suggesting that endogenous creatine synthesis plays a particularly critical role during growth and development. This aligns with prior research indicating an even higher ratio (10:1) in infants aged 0–12 months (Ostojic, 2024), further supporting the notion that endogenous creatine production is vital in early life. Additionally, our findings reveal an age-related decline in the availability of creatine precursors, with the highest levels observed in the youngest subgroup and the lowest in older adults from the NHANES III dataset. Although we estimated the maximal potential creatine synthesis from dietary glycine, arginine, and methionine under optimal conditions, several factors may limit actual endogenous production. These include: (1) amino acid bioavailability, which varies by protein source, food preparation methods, and individual gut health; (2) enzymatic regulation and feedback inhibition, which may constrain AGAT and GAMT activity; (3) competition for precursor amino acids with other metabolic pathways, such as the urea cycle (arginine), DNA methylation (methionine), and glutathione production (glycine); (4) metabolic constraints due to organ dysfunction, including kidney or liver disease; (5) hormonal influences, such as insulin, growth hormone, and age-related enzyme activity changes; and (6) dietary factors, such as insufficient intake of methyl donors (e.g., folate, vitamin B12) or overall protein deficiency. Consequently, even with high precursor intake, endogenous creatine synthesis may not reach its theoretical maximum, particularly in children, older adults, vegetarians, or individuals with metabolic disorders.

Our findings indicate that the intake of creatine precursors in our adult study population is approximately 40% higher than the levels previously reported in a U.S. adult cohort (Sarkar et al. 2021). This discrepancy may stem from methodological differences in dietary assessment tools, as well as technical challenges inherent in quantifying creatine intake in large-scale population studies (for a comprehensive discussion, see Ostojic, 2024). Notably, while the earlier study focused exclusively on adults, our analysis encompassed children and adolescents—age groups with distinct physiological demands and typically higher requirements and consumption of creatine-related amino acids. These differences in study populations likely contribute to the observed variation in intake estimates.

A critical gap in creatine research is the absence of direct measurements of endogenous synthesis across the general population. To date, no studies have employed stable isotope tracing, enzymatic activity assays, or nutrient depletion-repletion studies to quantify creatine biosynthesis in humans. The only direct estimate comes from a single-case supplementation study using labeled creatine, which inferred a turnover rate of 1.6 ± 0.5% per day under the assumption of first-order kinetics (Kan et al. 2006). However, this study did not account for potential suppression of endogenous synthesis by exogenous creatine intake (20 g/day). The extent of such inhibition for dietary creatine intake—typically 20 times lower than the supplementation dose—remains unknown. Brosnan et al. (2011) estimated daily endogenous creatine synthesis in young men at 8.3 mmol (15.5 mg/kg body weight for a 70-kg individual) based on dietary intake and creatinine excretion. However, this method assumes a steady-state balance, overlooking dynamic factors such as diet, physical activity, aging, and metabolic health. Additionally, creatinine excretion varies widely due to muscle mass, hydration status, and renal function (Ávila et al. 2025), further complicating estimates. Notably, our study estimated a median endogenous creatine synthesis rate of 28.9 mg/kg body weight per day in adult men, nearly double Brosnan’s estimate, likely due to our emphasis on dietary precursor intake. These discrepancies underscore the need for direct measurements of creatine biosynthesis under diverse dietary and physiological conditions.

While the NHANES III dataset provides a robust framework for evaluating dietary intake and estimated creatine synthesis, several methodological limitations should be acknowledged. The reliance on a single 24-hour dietary recall introduces potential recall bias and underreporting, particularly for creatine-rich foods. Our estimation method is based on correction factors from Brosnan et al. (2011), which do not account for individual variations in enzyme efficiency, cofactor availability, or genetic differences. Additionally, dietary creatine intake was derived from pre-existing databases, but food preparation and storage may alter actual creatine content. The lack of direct biochemical assessments, such as plasma guanidinoacetate levels or stable isotope tracers, limits the precision of our estimates. The cross-sectional design of NHANES III also prevents longitudinal assessments of creatine metabolism, making it difficult to establish causal relationships. Moreover, our model assumes a fixed proportion of dietary precursors is dedicated to creatine synthesis, without considering competition for these amino acids in other metabolic processes. Despite these limitations, our study provides novel insights into creatine precursor availability in a large, nationally representative cohort, emphasizing the need for future research incorporating biochemical validation, inter-individual variability, and longitudinal approaches.

The findings of this study hold important implications for public health nutrition, particularly in refining dietary recommendations aimed at optimizing creatine availability. Given creatine’s essential role in human physiology, a comprehensive understanding of its dietary sources—including direct intake and endogenous synthesis—is crucial for addressing potential deficiencies. Our results suggest that while endogenous synthesis significantly contributes to total creatine availability, this capacity declines with age. This underscores the need for targeted nutritional interventions, particularly among at-risk populations such as older adults, vegetarians, and individuals with metabolic disorders. Public health initiatives should consider strategies to ensure sufficient intake of creatine precursors through balanced diets or, where necessary, supplementation and food fortification (Ostojic 2021).

In addition, supplementation with dietary precursor amino acids has been reported to augment endogenous creatine synthesis by increasing substrate availability (Posey et al. 2024; Long et al. 2025). This strategy could be particularly beneficial for individuals with limited dietary creatine intake, such as vegetarians, older adults, or those with specific metabolic conditions. Observational data suggest that suboptimal dietary creatine intake in the general population may be associated with adverse health outcomes, including a higher risk of depression (Bakian et al. 2020), impaired female reproductive health (Ostojic et al. 2024), increased incidence of cardiovascular and hepatic disorders, and reduced cognitive function in older adults (Ostojic et al. 2021a, b). These findings support the rationale for exploring precursor amino acid supplementation as a potential nutritional intervention to bolster creatine status. However, this approach presents several limitations. The administration of precursor amino acids in excess may perturb metabolic homeostasis, potentially leading to unfavorable outcomes such as disrupted methylation pathways (methionine), increased nitrogen waste and urea production (arginine and glycine), or unintended stimulation of nitric oxide synthesis (arginine). Furthermore, the effectiveness of precursor supplementation may be constrained by the rate-limiting nature of AGAT, which is subject to negative feedback regulation by intracellular creatine levels. Thus, while precursor supplementation offers a plausible route to enhance endogenous creatine production, its clinical utility requires careful consideration of dosage, metabolic context, and individual variability. The potential concern regarding toxicity is also relevant in the context of excessive creatine intake. Nevertheless, the preponderance of evidence from both clinical and observational studies supports a favorable safety profile for exogenously administered creatine, even when consumed at relatively high doses. Comprehensive reviews of the literature, including the recent synthesis by Kreider and co-workers (2025), have consistently reported minimal adverse effects associated with creatine supplementation in both healthy and clinical populations. However, caution may be warranted for certain groups—such as individuals with pre-existing renal or gastrointestinal conditions—for whom excessive intake could pose greater risk. These findings reinforce the notion that, when used appropriately, creatine is a well-tolerated compound with a low risk of toxicity in the general population.

## Conclusion

Our study provides novel insights into the dietary availability of creatine-precursor amino acids and their contribution to endogenous creatine synthesis in the U.S. population. Our findings indicate that the estimated endogenous production of creatine from dietary glycine, arginine, and methionine is significantly greater than the direct intake of creatine from food sources, though this capacity declines with age. These results highlight the importance of adequate dietary intake of creatine precursors, particularly for populations at risk of reduced creatine synthesis, such as older adults. Future research should explore the metabolic constraints influencing endogenous creatine production and evaluate whether targeted dietary strategies or supplementation could help optimize creatine availability for health and performance benefits.

## Data Availability

The datasets backing the conclusions in this article are accessible in a publicly available repository as detailed below. The authors do not possess the data. The National Health and Nutrition Examination Survey data can be obtained from the National Center for Health Statistics at https://www.cdc.gov/nchs/nhanes/index. Further inquiries can be directed to the corresponding author.

## References

[CR1] Ávila M, Mora Sánchez MG, Bernal Amador AS, Paniagua R (2025) The metabolism of creatinine and its usefulness to evaluate kidney function and body composition in clinical practice. Biomolecules 15(1):41. 10.3390/biom15010041 39858438 10.3390/biom15010041 PMC11764249

[CR2] Bakian AV, Huber RS, Scholl L, Renshaw PF, Kondo D (2020) Dietary creatine intake and depression risk among U.S. Adults. Transl Psychiatry 10(1):52. 10.1038/s41398-020-0741-x 32066709 10.1038/s41398-020-0741-x PMC7026167

[CR4] Brosnan ME, Brosnan JT (2016) The role of dietary creatine. Amino Acids 48(8):1785–1791. 10.1007/s00726-016-2188-1 26874700 10.1007/s00726-016-2188-1

[CR3] Brosnan JT, da Silva RP, Brosnan ME (2011) The metabolic burden of creatine synthesis. Amino Acids 40(5):1325–1331. 10.1007/s00726-011-0853-y 21387089 10.1007/s00726-011-0853-y

[CR5] Delanghe J, De Slypere JP, De Buyzere M, Robbrecht J, Wieme R, Vermeulen A (1989) Normal reference values for creatine, creatinine, and carnitine are lower in vegetarians. Clin Chem 35(8):1802–1803 2758659

[CR6] Institute of Medicine (2005) Dietary reference intakes for energy, carbohydrate, fiber, fat, fatty acids, cholesterol, protein, and amino acids. National Academies, Washington, DC. 10.17226/10490

[CR7] Kan HE, van der Graaf M, Klomp DW, Vlak MH, Padberg GW, Heerschap A (2006) Intake of 13 C-4 creatine enables simultaneous assessment of creatine and phosphocreatine pools in human skeletal muscle by 13 C MR spectroscopy. Magn Reson Med 56(5):953–957. 10.1002/mrm.21068 17036281 10.1002/mrm.21068

[CR9] Korovljev D, Todorovic N, Stajer V, Ostojic SM (2021) Temporal trends in dietary creatine intake from 1999 to 2018: an ecological study with 89,161 participants. J Int Soc Sports Nutr 18(1):53. 10.1186/s12970-021-00453-1 34193199 10.1186/s12970-021-00453-1 PMC8247226

[CR10] Kreider RB, Gonzalez DE, Hines K, Gil A, Bonilla DA (2025) Safety of creatine supplementation: analysis of the prevalence of reported side effects in clinical trials and adverse event reports. J Int Soc Sports Nutr 22(sup1):2488937. 10.1080/15502783.2025.2488937 40198156 10.1080/15502783.2025.2488937 PMC11983583

[CR11] Long DW, Long BD, Nawaratna GI, Wu G (2025) Oral administration of L-arginine improves the growth and survival of sow-reared intrauterine growth-restricted piglets. Animals 15(4):550. 10.3390/ani15040550 40003032 10.3390/ani15040550 PMC11851912

[CR12] Lukaszuk JM, Robertson RJ, Arch JE, Moore GE, Yaw KM, Kelley DE, Rubin JT, Moyna NM (2002) Effect of creatine supplementation and a lacto-ovo-vegetarian diet on muscle creatine concentration. Int J Sport Nutr Exerc Metab 12(3):336–348. 10.1123/ijsnem.12.3.336 12432177 10.1123/ijsnem.12.3.336

[CR13] National Center for Health Statistics NHANES III, Survey Methods and Analytic Guidelines. Available at: https://wwwn.cdc.gov/nchs/nhanes/nhanes3/surveymethods.aspx

[CR19] Ostojic SM (2021) Creatine as a food supplement for the general population. J Funct Foods 83:104568. 10.1016/j.jff.2021.104568

[CR21] Ostojic SM (2022) Low tissue creatine: a therapeutic target in clinical nutrition. Nutrients 14(6):1230. 10.3390/nu14061230 35334887 10.3390/nu14061230 PMC8955088

[CR18] Ostojic SM (2025) Assessing dietary creatine intake in population studies: challenges and opportunities. Nutr Rev. (in press) 10.1093/nutrit/nuae155 10.1093/nutrit/nuae155 39441715

[CR20] Ostojic SM Establishing reference intakes for creatine in infants aged 0 to 12 months. Nutr Rev 2024 Sep 13:nuae124. Epub ahead of print. 10.1093/nutrit/nuae124 10.1093/nutrit/nuae124 39271173

[CR14] Ostojic SM, Forbes SC (2022) Creatine, a conditionally essential nutrient: Building the case. Adv Nutr 13(1):34–37. 10.1093/advances/nmab111 34662902 10.1093/advances/nmab111 PMC8803499

[CR16] Ostojic SM, Korovljev D, Stajer V (2021a) Dietary intake of creatine and risk of medical conditions in U.S. older men and women: Data from the 2017–2018 National Health and Nutrition Examination Survey. Food Sci Nutr 9(10):5746–5754. 10.1002/fsn3.2543 10.1002/fsn3.2543 PMC8498075 34646542

[CR15] Ostojic SM, Korovljev D, Stajer V (2021b) Dietary creatine and cognitive function in U.S. Adults aged 60 years and over. Aging Clin Exp Res 33(12):3269–3274. 10.1007/s40520-021-01857-4 33866527 10.1007/s40520-021-01857-4

[CR17] Ostojic SM, Stea TH, Ellery SJ, Smith-Ryan AE (2024) Association between dietary intake of creatine and female reproductive health: evidence from NHANES 2017–2020. Food Sci Nutr 12(7):4893–4898. 10.1002/fsn3.4135 39055234 10.1002/fsn3.4135 PMC11266896

[CR22] Posey EA, He W, Steele CC, Savell JW, Bazer FW, Wu G (2024) Dietary glycine supplementation enhances creatine availability in tissues of pigs with intrauterine growth restriction. J Anim Sci 102:skae344. 10.1093/jas/skae344 39513322 10.1093/jas/skae344 PMC11600959

[CR23] Sarkar TR, McNeal CJ, Meininger CJ, Niu Y, Mallick BK, Carroll RJ, Wu G (2021) Dietary intakes of amino acids and other nutrients by adult humans. Adv Exp Med Biol 1332:211–227. 10.1007/978-3-030-74180-8_12 34251646 10.1007/978-3-030-74180-8_12

[CR24] Todorovic N, Korovljev D, Stajer V, Jorga J, Ostojic SM (2022) Creatine consumption and liver disease manifestations in individuals aged 12 years and over. Food Sci Nutr 11(2):1134–1141. 10.1002/fsn3.3151 36789045 10.1002/fsn3.3151 PMC9922125

[CR25] Wu G (2020) Important roles of dietary taurine, creatine, carnosine, Anserine and 4-hydroxyproline in human nutrition and health. Amino Acids 52(3):329–360. 10.1007/s00726-020-02823-6 32072297 10.1007/s00726-020-02823-6 PMC7088015

[CR26] Wyss M, Kaddurah-Daouk R (2000) Creatine and creatinine metabolism. Physiol Rev 80(3):1107–1213. 10.1152/physrev.2000.80.3.1107 10893433 10.1152/physrev.2000.80.3.1107

